# Detection of *Leishmania* DNA in Phlebotomine Sand Flies in Tsatee, a Community in the Volta Region, Ghana

**DOI:** 10.1155/2023/1963050

**Published:** 2023-09-04

**Authors:** Seth Offei Addo, Emmanuel Kwame Amoako, Ronald Essah Bentil, Bright Agbodzi, Mba-tihssommah Mosore, Clara Yeboah, Naiki Attram, John Asiedu Larbi, Godwin Kwakye-Nuako, Dziedzom K. de Souza, Michael David Wilson, Daniel Adjei Boakye

**Affiliations:** ^1^Parasitology Department, Noguchi Memorial Institute for Medical Research, University of Ghana, Legon, Ghana; ^2^Department of Theoretical and Applied Biology, Kwame Nkrumah University of Science and Technology, Kumasi, Ghana; ^3^Department of Biomedical Sciences, School of Allied Health Sciences, College of Health and Allied Sciences, University of Cape Coast, Ghana

## Abstract

*Leishmania* parasites, which are spread by infected female sand flies, are the cause of the disease leishmaniasis. Although cutaneous leishmaniasis has been found to occur in the Volta Region, there is limited data on vector species and reservoirs. This study focused on the Tsatee community, in the South Dayi District of the Volta Region, and is aimed at identifying the sand fly fauna and detecting the presence of *Leishmania* DNA by the use of primers that target the conserved region of *Leishmania* spp. minicircle DNA of the parasite kinetoplast. The miniature light traps and hand aspirators provided by the Centers for Disease Control and Prevention (CDC) were used to collect outdoor and indoor sand flies for five months in a guinea woodland and semideciduous forest area. From the collections, 4,580 phlebotomine sand flies were obtained and identified, and females were examined for *Leishmania* DNA using PCR. The male flies were 1,202 (26.24%), non-blood-fed females were 3,321 (72.51%), and 57 (1.25%) were blood-fed females. It was observed that *Sergentomyia* species constituted 99.91% of the total collected sand flies with *S. africana* (76.77%) as the predominant species. *Phlebotomus rodhaini* (0.09%) was the only *Phlebotomus* species identified from the study area. From 283 non-blood-fed sand fly pools and 57 individual blood-fed species screened, *Leishmania* DNA was detected in 12 (4.24%) pools and 8 (14.04%) individuals, respectively. It was observed that *Leishmania* DNA was detected in all the sand fly species identified except *S. collarti*. This study reports the first detection of *Leishmania* DNA in *P. rodhaini* in Ghana, with an infection rate of 33.33% (95% CI, 1.23-88.32). The findings suggest that the role of *Phlebotomus* in disease transmission in the study area cannot be discounted. Future studies should include continuous surveillance, blood meal preferences, and vector competence of the various infected phlebotomine sand flies to create effective control measures.

## 1. Introduction

The disease cutaneous leishmaniasis (CL) causes skin lesions, mainly ulcers on exposed parts of the host, leaving scars. The transmission of most *Leishmania* responsible for CL is zoonotic [1]. About 350 million people globally are thought to be at risk of contracting the illness, which is thought to occur in 98 tropical, subtropical, and temperate regions [2]. Reported cases of CL in endemic communities of the Ho District suggest the presence of multiple *Leishmania* parasites including *Leishmania aethiopica* in humans [3], *Leishmania tropica*, and *Leishmania major* in *Sergentomyia* sand flies [4]. More recently, a new species causing infections in inhabitants of the Volta Region [5] has been characterised and named *Leishmania* (*Mundinia*) *chancei* [6]. With the spread of infections in communities within the Volta Region, it is not clear which specific *Leishmania* parasite is primarily responsible for infections and which vector is responsible for transmission.

Although *Phlebotomus* species are culprit vectors of *Leishmania* parasites, there is some evidence to suggest that some species of the genus *Sergentomyia* may also transmit the parasite [7–10]. The situation seems to be similar in Ghana as *L. tropica* and *L. major* DNA have been identified in pools of *Sergentomyia ingrami* and *Sergentomyia hamoni* [4]. *Leishmania major* DNA in pools of *Sergentomyia* species was closely related to that found in the human population of the same study area [11] suggesting *S. ingrami* as a possible vector due to its anthropophilic characteristics and abundance [12].

Suspected CL cases have been reported in Tsatee, South Dayi District of the Volta Region (personal communication). Tsatee shares similar climatic and environmental conditions with the Ho Municipality and is thus capable of supporting sand fly populations and transmission of *Leishmania*. In Ghana, the detection of a novel *Leishmania* species causing human infections [5, 6] further highlights a need to collect and identify potential vector(s) to determine whether the species previously considered as nonvectors can play a role in parasite transmission. Thus, this study focused on Tsatee, a community with suspected cases of CL, to identify sand fly species potentially involved in *Leishmania* transmission. The data obtained will be essential in selecting sand fly species for experimental studies to establish their possible roles in disease transmission in Ghana.

## 2. Methodology

### 2.1. Sand Fly Collection

The study was conducted in Tsatee (N 06°36.206′, E 000°14.689′), a community within the South Dayi District, in the Volta Region of Ghana. The district has a mix of semideciduous forest and guinea woodland as its vegetation. Human and animal shelters were randomly selected for sand fly collections using hand aspirators and CDC light traps, respectively, from June to August and October 2015. A total of 20 CDC light traps were used to collect outdoor sand flies while hand aspirators were used for collecting indoor resting sand flies. The collected sand flies were freeze killed, sorted by sex with the female and male sand flies preserved with silica gel and 70% ethanol, respectively, and sent to the laboratory for morphological identification using taxonomic keys [13] and further analysis (Supplementary Data 1).

### 2.2. Detection of *Leishmania* DNA in Sand Fly Samples

The identified female sand flies were pooled (1-20) based on the species, for DNA extraction using Qiagen DNA Mini Kit (Qiagen Inc., Hilden, Germany) following the manufacturer's instructions. The obtained genomic DNA was used as a template in PCR for the detection of *Leishmania* DNA, using oligonucleotide primers Mincr2-F (5′ GGG GAG GGG CGT TCT GCG AA 3′) and Mincr3-R (5′ CGC CCC CTA TTT TAC ACA ACC CC 3′). At a band size of 120 bp, these primers target the *Leishmania* spp. minicircle DNA of the parasite kinetoplast, conserved region [14, 15]. Positive controls (*L. major* and *L. tropica* DNA) obtained from culture and negative control (deionized water) were included in each PCR reaction. Each reaction was performed in a final volume of 25 *μ*l and contained 10× buffer, MgCl_2_ (25 mM), dNTP (0.2 mM of each), *Taq* DNA polymerase (1.25 units), nuclease-free water, and about 25 ng template DNA. The PCR was performed in a Gene Amp PCR system 9700 (Applied Biosystems, USA) with the cycling parameters as follows: 5 min at 94°C, 35 cycles for 30 sec at 94°C, 30 sec at 60°C, 1 min at 72°C, and a final extension for 3 min at 72°C. Ten microlitres of the PCR product was run on a 2% agarose gel for approximately 1 hour and 30 minutes at 100 volts. The gel was stained using ethidium bromide and visualised using Benchtop UVP Transilluminator (BioDoc-It® 210 Imaging System, Upland, California).

### 2.3. Data Analysis

The results obtained were documented using Microsoft Word and Excel 2013 and analysed to determine infections and infection rates in sand fly pools by making use of PoolScreen 2.0 (version 2.0.1, January 2002) [16].

## 3. Results

### 3.1. Distribution of Phlebotomine Sand Flies

A total of 4,580 phlebotomine sand flies were collected during the four-month sampling period which comprised of 1,202 (26.24%) males, 3,321 (72.51%) non-blood-fed females, and 57 (1.25%) blood-fed females. The majority of the sand flies were caught using the CDC light traps as opposed to the hand aspirators (Table 1).

### 3.2. Identification of Female Sand Flies

**Figure 1:**
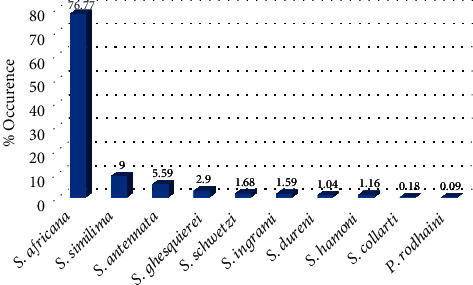
Distribution of sand fly species identified from the study area.

Of the 3,321 non-blood-fed females collected, 3,263 were successfully identified using taxonomic keys. Fifty-eight were either damaged or not in good condition for morphological identification. Outdoor collections recorded a total of 2,144 (65.71%) female sand flies compared to indoor collections which recorded 1,119 (34.29%). *Phlebotomus* were the least identified forming 0.09% (*n* = 3) as compared to 99.91% (*n* = 3260) of those belonging to the genus *Sergentomyia*. *Phlebotomus rodhaini* (0.09%) was the only species identified as belonging to the genus *Phlebotomus*. Of the genus *Sergentomyia*, *S. africana* (76.77%) was the predominant species (Figure 1).

A total of 57 blood-fed sand flies were collected from indoors (64.91%) and outdoors (35.09%) and morphologically identified as *S. africana* (57.89%), *S. similima* (22.81%), *S. antennata* (7.02%), *S. ingrami* 7.02%), and *S. schwetzi* (5.26%).

### 3.3. Detection of *Leishmania* DNA in the Identified Sand Fly Species

**Figure 2:**
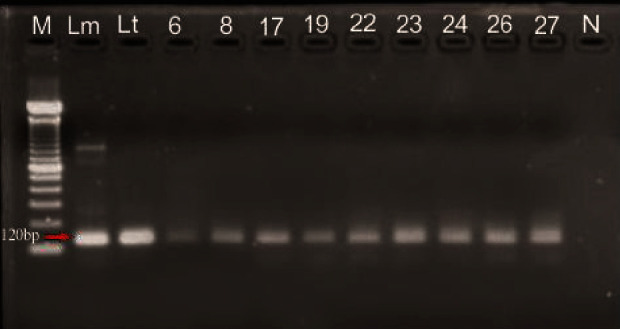
PCR gel showing the positive identification of *Leishmania* DNA in sand fly pools (lane M, 100 bp DNA ladder; Lm, *L. major*; Lt, *L. tropica*; lane 6, *S. africana*; lane 8, *S. similima*; lane 17, *S. antennata*; lane 19, *P. rodhaini*; lane 22, *S. dureni*; lane 23, *S. hamoni*; lane 24, *S. ghesquierei*; lane 26, *S. ingrami*; lane 27, *S. schwetzi*; and lane N, negative control).

A total of 283 pools of non-blood-fed sand flies were analysed, from which *Leishmania* DNA was detected in 12 (4.24%) pools (Figure 2). *Phlebotomus rodhaini* had an infection rate of 33.33% (95% CI, 1.23-88.32) (Table 2).

*Leishmania* DNA was detected in 10 (17.54%) out of the 57 individual blood-fed sand flies, with the infection rates recorded in the species as follows: *S. antennata* and *S. ingrami* 50% (95% CI, 7.72-92.28), respectively, *S. schwetzi* 33.33% (95% CI, 1.23-88.32), *S. africana* 12.12% (95% CI, 3.21-28.30), and *S. similima* 7.69% (95% CI, 0.25-33.81).

## 4. Discussion

The first outbreak of cutaneous leishmaniasis (CL) occurred in the Ho Municipality of the Volta Region in 1999 which is further south of the West African CL belt [12]. The disease has persisted since then and is currently spreading to previously nonendemic districts.

This study was carried out outside the Ho District at Tsatee in the South Dayi District, where CDC light traps and hand aspirators were used. However, the results obtained collaborate with the higher preponderance of *Sergentomyia* species with few *Phlebotomus* species found by previous studies in the Ho District. Since 1999, various epidemiology, entomology, and parasitology studies have been carried out within the Ho District. These studies involved the identification of the possible vectors and the *Leishmania* species responsible for the disease in the affected communities. Three trapping methods (CDC light traps, sticky traps, and hand aspirators) used in these studies had previously caught *Sergentomyia* species in predominantly large numbers and very few *Phlebotomus* species suggesting that the latter may not be involved in the disease transmission [4, 12]. In Ghana, the specific vectors responsible for *Leishmania* transmission remain uncertain, reservoir hosts are still unknown, and treatment for infections is not in place. Because vaccines are not available for the prevention of cutaneous leishmaniasis infection, intervention measures to control the infection should be put in place to prevent or reduce the rate of sand fly bites and thus reduce the burden of the disease [17]. It is also essential to understand the vectors involved, taking into account factors such as their feeding habits and transmission periods to establish prevention and control strategies [18].

In this study, a total of 99.91% of the species identified were *Sergentomyia* which is similar to the species composition in the Ho District found by Fryauff et al. [11]. Furthermore, *Sergentomyia africana* (76.69%) was the most predominant species. *Phlebotomus* species, specifically *P. rodhaini* (0.09%), identified were in low numbers which is also consistent with the results obtained by Fryauff et al. [11]. However, the low occurrence does not minimize the importance of the vector species. For instance, in eastern Sudan, a study carried out indicated *P. rodhaini* to be a potential vector of *L. donovani* despite their occurrence in small numbers [19]. The author further proposed that *P. rodhaini* was a possible vector responsible for *L. donovani* transmission between animal reservoir hosts but did not infect humans. Also in parts of West Africa, *P. rodhaini* is a vector for *L. major* [20].

All the blood-fed sand flies collected were *Sergentomyia* with *S. africana* (57.89%) being the dominant species. Man-biting species, *S. schwetzi* as described by Boakye et al. [21], was also identified but in low numbers. The predominance of *Sergentomyia* species suggests that they may play a role in the transmission of *Leishmania* parasites within the present study area and the Volta Region at large. Whether they transmit zoonotic leishmaniasis was not determined by the present study.

*Sergentomyia* species transmit reptilian *Leishmania* [22]. However, the findings from certain studies suggest that *Sergentomyia* species could vector human *Leishmania*. For example, a study conducted in Kenya supports the role of *Sergentomyia* species in the transmission of mammalian-infecting *Leishmania* parasites [23]. The authors found an infection rate of 1% in *S. ingrami* and also observed that *L. major* isolated from the guts of infected females produced lesions when inoculated into BALB/c mice. The findings, coupled with a large number of amastigotes found in prepared smears of the lesions, led the authors to suggest a role of *S. ingrami* in transmitting *L. major* in the study area [23]. In Mali and Kenya, *L. major* DNA was detected in *S. darlingi* and *S. garnhami*, respectively [8, 24]. In this study, *Leishmania* DNA was detected in all the trapped non-blood-fed sand fly species except *S. collarti* unlike in the Ho District where parasite DNA was detected in only pools of *S. ingrami* and *S. hamoni* [4]. Amongst the 57 individual blood-fed sand flies, *Leishmania* DNA was detected in all five identified species. *S. ingrami* and man-biting species *S. schwetzi* were trapped in the dwellings of inhabitants in the study area. Even though *Leishmania* DNA was detected in *Sergentomyia* species, that is not enough evidence to consider them vectors. Since infections from blood meals may last in nonvectors for a while but ultimately do not result in established transmissible disease, harbouring the parasites does not inevitably make them vectors [25]. Furthermore, a study showed that even though *S. schwetzi* was infected with *L. major*, complete development of the parasite did not occur and was not transmitted to a host postfeeding [26]. Studies have also reported the refractoriness of *Sergentomyia* to *Leishmania* parasites [10, 22, 27]. Establishing colonies of *Sergentomyia* species in Ghana will be necessary for infection experiments aimed at observing their potential to house the complete development of *Leishmania* parasites and further transmit them to a host.

For the first time in Ghana, *Leishmania* DNA was detected in a single unfed *P. rodhaini*, thus an infection rate of 33.33%. This suggests that although *P. rodhaini* occurs in low numbers, it can be highly infected, hence can, therefore, be considered as a potential vector of *Leishmania* parasite transmission in the study area. *P. rodhaini* were collected outdoors which makes it uncertain as to whether the parasite transmission is occurring from reservoir hosts to humans or human to human.

With the discovery of a new *Leishmania* species [5], more questions rather than answers are being raised. It is not clear which *Leishmania* species are responsible for infections in communities of the Volta Region. Further investigations will be essential to fully determine the different *Leishmania* parasites in the region and identify the vector(s) responsible for infections.

## 5. Conclusions

The present study has confirmed the dominance of *Sergentomyia* species in previous studies in CL endemic areas of the Volta Region and the relatively low occurrence of *Phlebotomus* species. Although *Sergentomyia* species are considered to be nonvectors for human pathogenic *Leishmania* species, some harboured *Leishmania* DNA indicating that they may be involved in the disease transmission. Further experiments are however required to demonstrate their ability to accommodate the complete development of *Leishmania* parasites and further transmit to a suitable host. This study also reports for the first time in Ghana the detection of *Leishmania* DNA in *P. rodhaini*. The findings suggest *P. rodhaini* as a potential vector of cutaneous leishmaniasis at Tsatee in the South Dayi District.

## Figures and Tables

**Table 1:** Distribution of sand flies trapped using CDC light trap and hand aspirator.

Trapping methods	NBF females	BF females	Males	Total (%)
CDC light trap	2192	20	994	3,206 (70%)
Aspirator	1129	37	208	1,374 (30%)
Total	3321	57	1202	4,580

NBF: non-blood-fed female sand flies; BF: blood-fed female sand flies.

**Table 2:** Infection rates in non-blood-fed sand flies.

Species	Total pools	Minimum pool size	Maximum pool size	No. of positive	Infection rates (95% CI)
*S. africana*	138	1	20	4	0.16 (0.41-0.42)
*S. antennata*	25	2	20	1	0.63 (0.02-3.20)
*S. dureni*	10	1	8	1	3.30 (0.1-16.04)
*S. ghesquierei*	15	2	19	1	1.05 (0.03-5.30)
*S. hamoni*	14	1	7	1	2.60 (0.08-12.80)
*S. ingrami*	19	1	9	1	1.92 (0.06-9.52)
*S. schwetzi*	19	1	7	1	1.80 (0.06-9.01)
*S. similima*	34	1	20	1	0.32 (0.01-1.60)
*P. rodhaini*	3	1	1	1	33.33 (1.23-88.32)
*S. collarti*	6	1	1	0	0
Total	283			12	

CI: confidence interval.

## Data Availability

This article contains all the data necessary to support the findings.

## Supplementary Materials

Supplementary Materials Supplementary material about the morphological identification of female phlebotomine sand flies in this study has been attached as Supplementary Data 1.

## References

[1] Reithinger R., Dujardin J.-C., Louzir H., Pirmez C., Alexander B., Brooker S. (2007). Cutaneous leishmaniasis. *Infectious Diseases*.

[2] Alvar J., Vélez I. D., Bern C. (2012). Leishmaniasis worldwide and global estimates of its incidence. *PLoS One*.

[3] Kwakye-Nuako G. (2010). *Leishmania aethiopica*: the unusual etiologic agent of cutaneous leishmaniasis in Ho District of the Volta Region of Ghana. *International Journal of Infectious Diseases*.

[4] Nzelu C. O., Kato H., Puplampu N. (2014). First detection of *Leishmania tropica* DNA and *Trypanosoma* species in *Sergentomyia* sand flies (Diptera: Psychodidae) from an outbreak area of cutaneous leishmaniasis in Ghana. *PLoS Neglected Tropical Diseases*.

[5] Kwakye-Nuako G., Mosore M.-T., Duplessis C. (2015). First isolation of a new species of *Leishmania* responsible for human cutaneous leishmaniasis in Ghana and classification in the *Leishmania enriettii* complex. *International Journal for Parasitology*.

[6] Kwakye-Nuako G., Mosore M. T., Boakye D., Bates P. A. (2023). Description, biology, and medical significance of *Leishmania* (*Mundinia*) *chancei* n. sp. (Kinetoplastea: Trypanosomatidae) from Ghana and *Leishmania* (*Mundinia*) *procaviensis* n. sp. (Kinetoplastea: Trypanosomatidae) from Namibia. *The Journal of Parasitology*.

[7] Berdjane-Brouk Z., Koné A. K., Djimdé A. A. (2012). First detection of *Leishmania major* DNA in *Sergentomyia* (*Spelaeomyia*) *darlingi* from cutaneous leishmaniasis foci in Mali. *PLoS One*.

[8] Campino L., Cortes S., Dionsio L., Neto L., Afonso M. O., Maia C. (2013). The first detection of *Leishmania major* in naturally infected *Sergentomyia minuta* in Portugal. *Memorias Do Instituto Oswaldo Cruz*.

[9] Kanjanopas K., Siripattanapipong S., Ninsaeng U. (2013). Sergentomyia (*Neophlebotomus*) *gemmea*, a potential vector of *Leishmania siamensis* in southern Thailand. *BMC Infectious Diseases*.

[10] Maia C., Depaquit J. (2016). Can *Sergentomyia* (Diptera, Psychodidae) play a role in the transmission of mammal-infecting *Leishmania*?. *Parasite*.

[11] Fryauff D. J., Hanafi H. A., Klena J. D. (2006). ITS-1 DNA sequence confirmation of *Leishmania major* as a cause of cutaneous leishmaniasis from an outbreak focus in the Ho District, southeastern Ghana. *The American Journal of Tropical Medicine and Hygiene*.

[12] Kweku M. A., Odoom S., Puplampu N. (2011). An outbreak of suspected cutaneous leishmaniasis in Ghana: lessons learnt and preparation for future outbreaks. *Global Health Action*.

[13] Abonnenc E. (1972). Les phlébotomes de la région Éthiopienne (Diptera, Psychodidae). *Cahiers de l'ORSTOM, série Entomologie médicale et Parasitologie*.

[14] Degrave W., Fernandes O., Campbell D., Bozza M., Lopes U. (1994). Use of molecular probes and PCR for detection and typing of Leishmania - a mini-review. *Memórias do Instituto Oswaldo Cruz*.

[15] da Silva E. S., Gontijo C. M., Pacheco Rda S., Brazil R. P. (2004). Diagnosis of human visceral leishmaniasis by PCR using blood samples spotted on filter paper. *Genetics and Molecular Research*.

[16] Katholi C. R., Unnasch T. R. (2006). Important experimental parameters for determining infection rates in arthropod vectors using pool screening approaches. *The American Journal of Tropical Medicine and Hygiene*.

[17] Selmane S. (2016). *The Impact of Climate Conditions on Cutaneous Leishmaniasis Incidence T*.

[18] Dantas-Torres F., Otranto D. (2016). Best practices for preventing vector-borne diseases in dogs and humans. *Trends in Parasitology*.

[19] Elnaiem D. E. A., Hassan H. K., Osman O. F., Maingon R. D., Killick-Kendrick R., Ward R. D. (2011). A possible role for *Phlebotomus* (*Anaphlebotomus*) *rodhaini* (Parrot, 1930) in transmission of *Leishmania donovani*. *Parasites and Vectors*.

[20] Anderson J. M., Samake S., Jaramillo-Gutierrez G. (2011). Seasonality and prevalence of *Leishmania major* infection in *Phlebotomus duboscqi* neveu-lemaire from two neighboring villages in Central Mali. *PLoS Neglected Tropical Diseases*.

[21] Boakye D. A., Wilson M., Kweku M. (2005). A review of leishmaniasis in West Africa. *Ghana Medical Journal*.

[22] Sadlova J., Dvorak V., Seblova V., Warburg A., Votypka J., Volf P. (2013). *Sergentomyia schwetzi* is not a competent vector for *Leishmania donovani* and other *Leishmania* species pathogenic to humans. *Parasites & Vectors*.

[23] Mutinga M. J., Kyai F. M., Omogo D. M. (1986). Investigations on the epidemiology of leishmaniases in Kenya—I. Studies on vectors of *Leishmania major* in Marigat, Baringo District, Kenya. *International Journal of Tropical Insect Science*.

[24] Mutinga M. J., Massamba N. N., Basimike M. (1994). Cutaneous leishmaniasis in Kenya: Sergentomyia garnhami (Diptera Psychodidae), a possible vector of *Leishmania major* in Kitui District: a new focus of the disease. *East African Medical Journal*.

[25] Ready P. D. (2013). Biology of phlebotomine sand flies as vectors of disease agents. *Annual Review of Entomology*.

[26] Lawyer P. G., Ngumbi P. M., Anjili C. O. (1990). Development of *Leishmania major* in *Phlebotomus duboscqi* and *Sergentomyia schwetzi* (Diptera: Psychodidae). *The American Journal of Tropical Medicine and Hygiene*.

[27] Sadlova J., Homola M., Myskova J., Jancarova M., Volf P. (2018). Refractoriness of *Sergentomyia schwetzi* to *Leishmania* spp. is mediated by the peritrophic matrix. *PLoS Neglected Tropical Diseases*.

