# Supplementary material for: Detection of Leishmania DNA in Phlebotomine Sand Flies in Tsatee, a Community in the Volta Region, Ghana
Source: Biomed Res Int. 2023 Sep 4;2023:1963050. doi: 10.1155/2023/1963050 (PMC10495235; doi:10.1155/2023/1963050)
Supplement: Supplementary Materials — Supplementary material about the morphological identification of female phlebotomine sand flies in this study has been attached as Supplementary Data 1. [file 1963050.f1.docx]

**Detection of *Leishmania* DNA in Phlebotomine sand flies in Tsatee, a community in the Volta Region, Ghana**

Seth Offei Addo^1,2*^, Emmanuel Kwame Amoako^1^, Ronald Essah Bentil^1^, Bright Agbodzi^1^, Mba-tihssommah Mosore^1^, Clara Yeboah^1^, Naiki Attram^1^, John Asiedu Larbi^2^, Godwin Kwakye-Nuako^3^, Dziedzom K. de Souza^1^, Michael David Wilson^1^, Daniel Adjei Boakye^1^.

^1^Parasitology Department, Noguchi Memorial Institute for Medical Research, University of Ghana, Legon, Ghana.

^2^Department of Theoretical and Applied Biology, Kwame Nkrumah University of Science and Technology, Kumasi, Ghana

^3^Department of Biomedical Sciences, School of Allied Health Sciences, College of Health and Allied Sciences, University of Cape Coast, Ghana.

* Corresponding author: Seth Offei Addo ([sethaddo40@gmail.com](mailto:sethaddo40@gmail.com))

**Identification of Female Phlebotomine sand flies**

To identify the female sand flies, each was dissected under a dissecting microscope using a pair of dissecting pins. The head and last three segments of the abdomen were removed and placed in a labelled 0.2 ml sterile microtube containing 2 drops of clearing medium (chloral hydrate and phenol) for about 24 hours. The remaining thorax and upper abdominal segments were kept in corresponding 0.2 ml sterile tubes for molecular analysis. The head and last three segments of the abdomen were further mounted on a glass slide and a drop of mounting medium (8 g Arabic gum, 70 g chloral hydrate, 10 ml distilled water, 5 ml glycerin and 3 ml glacial acetic) was added.

Making sure the head was placed with the proboscis facing upward and the last abdominal segments in a lateral position, a glass coverslip was used to cover the sample. The slide with the fixed sand fly was allowed to clear and dry using Slide Warmer Model XH-2001 at about 55-60°C for about 2 weeks and then observed under an optical microscope for species identification.

Sand flies were identified and grouped into species using taxonomic keys (Abonnenc, 1972). Morphological features used in the identification of species included the presence of cibarium, cibarial teeth, pharynx and spermatheca. An Olympus BH-2 mounted camera connected to a monitor utilizing software was used to capture images of the features of different species.

In the identification process, three main features were considered including the cibarium, pharynx and spermatheca. The cibarium is a structure that lies between the pharynx and the proboscis in the head. Present in the cibarium are cibarial teeth which are unique for various species. The pharynx also located in the head and usually a bottle or lamp-glass shaped, is a posterior continuation of the cibarium consisting of a dorsal and ventral plate. The spermatheca, found in only the females, varies in size and shape for different species. *Phlebotomus* species have a cibarium which lacks cibarial teeth and a pigmented patch whereas *Sergentomyia* species have both the teeth and pigment patch present
